# Mesoporous Carbons from Polysaccharides and Their Use in Li-O_2_ Batteries

**DOI:** 10.3390/nano10102036

**Published:** 2020-10-15

**Authors:** María Uriburu-Gray, Aránzazu Pinar-Serrano, Gokhan Cavus, Etienne Knipping, Christophe Aucher, Aleix Conesa-Cabeza, Amro Satti, David Amantia, Sandra Martínez-Crespiera

**Affiliations:** 1 LEITAT Technological Centre, Applied Chemistry and Materials Department, C/Pallars, 185, 08005 Barcelona, Spain; muriburu@leitat.org (M.U.-G.); apinar@leitat.org (A.P.-S.); asatti@leitat.org (A.S.); damantia@leitat.org (D.A.); 2 LEITAT Technological Centre, Energy and Engineering Department, C/de la Innovació, 2, 08225 Terrassa (Barcelona), Spain; gcavus@leitat.org (G.C.); eknipping@leitat.org (E.K.); caucher@leitat.org (C.A.); 3 LEITAT Technological Centre, Scientific and Technical S-ervices Division, C/de la Innovació, 2, 08225 Terrassa (Barcelona), Spain; aconesa@leitat.org

**Keywords:** starch, chitosan, polysaccharide, amylose, ionic liquid, carbon, Starbon^®^, mesoporosity, Li-O_2_ battery cell

## Abstract

Previous studies have demonstrated that the mesoporosity of carbon material obtained by the Starbon^®^ process from starch-formed by amylose and amylopectin can be tuned by controlling this ratio (the higher the amylose, the higher the mesoporosity). This study shows that starch type can also be an important parameter to control this mesoporosity. Carbons with controlled mesoporosity (V_meso_ from 0.1–0.7 cm^3^/g) have been produced by the pre-mixing of different starches using an ionic liquid (IL) followed by a modified Starbon^®^ process. The results show that the use of starch from corn and maize (commercially available Hylon VII with maize, respectively) is the better combination to increase the mesopore volume. Moreover, “low-cost” mesoporous carbons have been obtained by the direct carbonization of the pre-treated starch mixtures with the IL. In all cases, the IL can be recovered and reused, as demonstrated by its recycling up to three times. Furthermore, and as a comparison, chitosan has been also used as a precursor to obtain N-doped mesoporous carbons (5.5 wt% N) with moderate mesoporosity (V_meso_ = 0.43 cm^3^/g). The different mesoporous carbons have been tested as cathode components in Li-O_2_ batteries and it is shown that a higher carbon mesoporosity, produced from starch precursor, or the N-doping, produced from chitosan precursor, increase the final battery cell performance (specific capacity and cycling).

## 1. Introduction

Every year, there is an increasing demand of energy due to growing populations. Most of the consumed energy still comes from fossil fuel (carbon, oil, and natural gas). Their combustion is causing the greenhouse gas emissions (e.g., carbon dioxide, methane, hydrogen sulphide, nitrous oxide, etc.), contributing to climate change. In order to mitigate global climate change, the transition from fossil fuel’s energy to zero-emission energy is necessary and is only possible with the development of sustainable materials and technologies. 

The production of porous carbon materials from natural and biomass sources such as polysaccharides (starch [1,2,3], alginic acid [4], chitosan [5], and cellulose), lignin, tannins [6,7], seeds [4], green phenolic resins [8,9], etc. has been widely investigated. Porous carbon materials have been used for different applications such as catalysis, energy storage, gas adsorption, or water purification. As such, these materials play an important role as supports in catalytic reactions due to their high surface area, hierarchical porosity, chemical inertness, and electrical conductivity [10]. Some of these properties can be modulated during the synthesis of the material, e.g., an increased conductivity can be achieved at higher calcination temperatures since graphitization increases with temperature. In addition, the activation of the carbon and, therefore, the formation of micropores can be achieved by physical (e.g., CO_2_, steam) or chemical (e.g., KOH) methods [11,12]. Different applications were found in catalysis, working both as a catalyst (carbocatalysis [13]) and as supporting material during the catalytic electrochemical reactions (e.g., Oxygen Reduction Reaction (ORR) [14,15], Carbon Dioxide Reduction Reaction (CO_2_RR) [16]). Furthermore, high conductivity carbonaceous materials have been investigated for energy storage applications using carbon as electrodes in systems such as super-capacitors, fuel cells, or rechargeable batteries (Li-ion [17], Na-ion [18], Li-S [19], and Li-air [20]).

In 2007, White, Clark, Budarin, and MacQuarrie, from the University of York, designed and patented a method called Starbon^®^ process [2,21] for the preparation of mesoporous carbons from starch. This method involved a first dispersion of the starch in water, and, after gelatinization, retrogradation and dehydration processes of an expanded dried starch was obtained. Finally, the expanded dried starch was carbonized under inert atmosphere to obtain the mesoporous carbons.

Starch is one of the major polysaccharide sources in nature, present in roots (e.g., ginger), tubers (e.g., potato), cereals (e.g., corn, oats, wheat, rice), and fruits (e.g., banana). It is mainly composed by amylose and amylopectin, and proteins and lipids in minor quantities. Amylose and amylopectin are polysaccharides formed by glucose units joined by glycosidic bonds in a lineal and branched structure, respectively, depending on the source of the starch. The ratio amylose: amylopectin varies and so are the physico-chemical properties of the material. Several studies demonstrated that the higher the amylose content is in starch, the higher the porosity of the carbonized material is [12].

Ionic liquids (ILs) are ionic and non-molecular salts that melt below 100 °C. Normally, they are composed by an organic cation and an organic or inorganic anion, and they are very interesting because of their distinctive properties, such as low vapor pressure, high thermal stability, recyclability, conductivity, and so on [22]. Due to the low vapor pressure, and good recyclability, ILs have been widely used as green solvents because they do not contribute to the emission of volatile organic compounds (VOCs) to the atmosphere [23]. Furthermore, ILs present many molecular interactions that make them powerful solvents for different species, as reported in 2002 by Rogers et al., where they showed very good results dissolving cellulose with 1-Butyl-3-methylimidazolium chloride (BMIMCl) [24]. BMIMCl interacts with the hydrogen bonds of the cellulose promoting the dissolution of the polysaccharide [22]. 

In this work, we demonstrate the preparation of carbon materials with controlled mesoporosity from polysaccharides using a process based on an IL pre-treatment (with recovery and reuse of the IL), which is followed by a modified Starbon^®^ process (with ultrasonication instead of a microwave treatment at high temperatures). The mesoporosity is influenced not only by the amylose content but also by the starch type. In this way, the starch mixture of Maize starch (commercial starch from maize) and Hylon VII starch (commercial starch from corn) with 60 wt% of amylose results in a higher mesoporosity than the pure Hylon VII starch (commercial starch from corn) with 70 wt% of amylose. At lower amylose content (less than 45 wt%), the higher mesoporosity is obtained with the mixture of Maize and Hylon V. The recovery and reuse of the IL is studied up to three times with minor changes in the final mesoporosity. Additionally, we show a low-cost process for the production of mesoporous carbons from starch by avoiding the need to perform gelation, retrogradation, and freeze-drying (a saving of four days) as required in the Starbon^®^ process. Finally, we demonstrate that the performance of these carbons as cathode materials in Li-O_2_ batteries is greatly influenced by their mesoporosity, resulting in higher capacity and cycling for the carbons with a higher mesoporosity or N-doping [25,26,27].

## 2. Materials and Methods 

### 2.1. Materials and Equipment Used

The starches maize (M), Hylon V (HV), and Hylon VII (HVII) were obtained from Ingredion^®^ (Manchester, UK). Pure amylose, pure amylopectin, chitosan (MMW), potassium iodide (KI), dimethyl sulfoxide (DMSO) 99.9% *v*/*v*, and para-toluenesulfonic acid monohydrate (p-TSA) were purchased from Sigma-Aldrich (Darmstadt, Germany). The ionic liquid (IL) 1-butyl-3-methylimidazolium chloride 99% (BMIMCl) was purchased from abcr (Karlsruhe, Germany). Ethanol 96% v/v, iodine (I_2_), and sodium hydroxide (pellets) were purchased from Scharlab (Barcelona, Spain). Tert-butanol (TBA) was obtained from Honeywell (Charlotte, NC, USA).

The amylose content in the commercially available starches was determined by a colorimetric method measuring the absorbance at 600 nm of a complex formed between amylose and iodine by UV-Vis spectrophotometry. The equipment used was Jasco V-630 UV-Vis spectrophotometer (Jasco Inc., Easton, MD, USA).

Fourier-Transform Infrared-Attenuated Total Reflection (FTIR-ATR) spectroscopy was performed using an Agilent Cary 630 FTIR (Agilent Technologies Inc., Santa Clara, CA, USA), after 32 scans with a spectral resolution of 4 cm^−1^ and a spectral range from 4000 to 500 cm^−1^. Proton nuclear magnetic resonance (^1^HNMR) spectroscopy was performed using a Bruker Avance DPX spectrometer (Bruker Corporation, Billerica, MA, USA) of 250 MHz (5.8 T) equipped with an automatic sample changer Bruker BACS-60 and a probe 1H/13C/31P.

Thermogravimetric analysis (TGA) was performed with a TA instrument Thermogravimetric Analyzer Q500 (Waters Corporation, Milford, MA, USA). The samples were loaded in flat platinum pans and the measurements were taken under a heating rate of 10 °C/min from room temperature to 900 °C.

Gas Permeation Chromatography (GPC) was performed using an Agilent 1200 Infinity GPC (Agilent Technologies Inc., Santa Clara, CA, USA) equipped with a 390-LC Multi Detector Suite.

High Resolution Scanning Electron Microscopy (HR-SEM) was performed using a Carl Zeiss MERLIN^®^ Field Emission-Scanning Electron Microscopy (FE-SEM) microscope (Carl Zeiss AG, Oberkochen, Germany) equipped with a GEMINI^®^ II column, and High-Resolution Transmission Electron Microscopy (HR-TEM), using a Jeol JEM-2011 scanning microscope (JEOL Ltd., Akishima, Tokyo, Japan).

Nitrogen adsorption and desorption isotherms were measured using a Nova 2200e Surface Area and Pore Analyzer from Quantachrome Instruments (Anton Paar, Graz, Austria). The surface area was determined by the application of multi-point Brunauer-Emmett-Teller (BET) equation and the pore size was calculated according to the Barrett, Joyner, and Halenda (BJH) average pore diameter equation, D = 4V_BJH_/SA_BJH_.

Quantitative Nitrogen determination was measured by an Elemental Analyzer Eurovector EuroEA3000 (EuroVector S.p.A., Milan, Italy).

The micro-Raman spectra were recorded using a 532 nm laser excitation line with a dispersive spectrometer Jobin-Yvon LabRam HR 800 (Horiba Ltd., Kyoto, Japan), coupled to an optical microscope Olympus BXFM (Olympus Corporation, Tokyo, Japan).

### 2.2. Determination of Real Amylose Content in Commercially Available Starches

In order to determine the real content of amylose of the obtained commercial starches [28,29], a calibration graph was first obtained. For that, a 100 mL of iodine solution was prepared containing KI 6.5 mM and I_2_ 2.5 mM in distilled water (this solution must be stored in darkness at 4 °C). Then, different starch solutions containing different ratios of pure amylose and amylopectin (0, 10, 20, 30, 40, 50, 60, 70, 80, 90, and 100 wt% amylose) were prepared by dissolving 20 mg of mixture into 8 mL of DMSO and heated for 15 min at 85 °C in a water bath. The solutions were allowed to cool and then diluted to 25 mL with distilled water. After, a solution was prepared by the addition of 40 mL of distilled water to 1 mL of the starch precursor solution, which was followed by the addition of 5 mL of the iodine precursor solution and, finally, diluted to 50 mL of distilled water. The formation of an amylose-iodine complex was measured by the Ultraviolet-Visible (UV-VIS) absorbance at 600 nm. The calibration graph obtained was used to calculate the real amylose content of the commercially available starches. For this, previously, the starches were defatted with a treatment with 120 mL 75% n-propanol at 85 °C for 7 h in a Soxhlet extractor to eliminate the lipids. Then, the determination of amylose in the commercial starches was carried out by the same procedure described above but adding 20 mg of the starch.

### 2.3. Preparation of Mesoporous Carbons from a Modified Starbon^®^ Based Process

#### 2.3.1. Modified Starbon^®^ Based Process

This process was carried out by dispersing a 10 wt% of the starch mixture with distilled water under strong magnetic stirring for 1 h. Then, the dispersion was ultrasonicated for 15 min (2200 J) using a tip (Ultrasonic processor Sonics VCX750 equipped with a solid 13-mm probe 630-0219, Sonics & Materials, Newtown, CT, USA) in a pulse mode of 1 s. After cooling at room temperature, the gel was maintained at 5 °C for 2 days to allow the retrogradation of the structure. After that time, 30 wt% of TBA with respect to the total water content was added to the gel and kept for 1 h in a rotatory mixer at lower speed rotation (5 rpm). Then, 1 wt% of p-TSA with respect to starch was added and kept for 1 h under rotation. The gel was then frozen with liquid nitrogen and dried for 2 days in a freeze-dryer (Scanvac Coolsafe 100-9 Pro Freeze-dryer, LaboGene A/S, Allerød, Denmark) at 0.02 mbar and room temperature. After freeze-drying, an expanded dried gel was obtained. The carbonization process was carried out under inert atmosphere (argon) in a multi-stage process from room temperature to 800 °C for 24 h in a laboratory chamber furnace (CWF 12/23, Carbolite Inc., Sheffield, UK).

#### 2.3.2. Modified Starbon^®^ Based Process with a Previous Treatment of the Starches with the IL

In the first step of this process, the starches were dispersed in an IL. For this, BMIMCl was heated at 95 °C under mechanical stirring until completely melted. Then, 5 g of the starch or the mixture were added to the IL (20 wt% of starch) and stirred for 6 h at 95 °C by forming a viscous white dispersion. After that, the starch or the mixture was quantitatively separated from the IL using several washing steps. In the first step, 75 mL of distilled water was added dropwise, the dispersion was heated to 70 °C, and 150 mL of ethanol were added dropwise to precipitate the starch. The mechanical agitation was maintained overnight, and the next day the precipitate was separated by centrifugation at 4500 rpm for 10 min, which was followed by several washings with ethanol until complete elimination of IL, detected by FTIR at 1560 cm^−1^. Finally, the precipitate was separated by vacuum filtration and dried overnight at 80 °C and 60 mbar, obtaining a completely white powder.

In the following step, the Starbon^®^ based process was performed as explained before in Section 2.3.1, and, finally, carbonized at 800 °C or 1000 °C by the same procedure.

### 2.4. Preparation of Mesoporous Carbons with the “Low-Cost” Process

This process involves the previous preparation of the starch or mixture in the IL, as explained in Section 2.3.2, but without the gelatinization and freeze-drying process. Therefore, after the IL pre-treatment and the addition of 1 wt% of p-TSA with respect to the starch, the sample was directly carbonized at 800 °C, following the same conditions as explained before.

### 2.5. IL Recyclability

After the starch separation in the process described in Section 2.3.2, a liquid phase of IL, ethanol, and water was obtained. In order to recover and reuse the IL, the ethanol and water were first evaporated at 5 mbar and 95 °C. Afterward, the recovered IL was treated with molecular sieves (MS) of 4 Å, previously activated at 200 °C and 2 mbar, and left spinning in a rotatory tube mixer for two days. The solid IL was recovered after melting at 100 °C and filtrating to separate it from the molecular sieves.

### 2.6. Preparation of N-Doped Mesoporous Carbon from Chitosan

The preparation of N-doped mesoporous carbon from chitosan was carried out by dissolving 2 g of chitosan into 80 g of distilled water, acidified with 1 mL of acetic acid. The mixture was stirred for 1 h and then it was maintained for 1 day at 5 °C for the gelation process. Then the gel was precipitated into 100 mL of NaOH 3 M and washed with distilled water until complete neutralization. The precipitate was separated and 30 wt% TBA was added and kept under rotation for 1 h. The mixture was freeze-dried for 2 days before carbonization at 1000 °C under argon, following the same conditions.

### 2.7. Cell Assembly and Electrochemical Characterization

All electrochemical tests were carried out in the same reference battery cell system. An ECC-Air cell (EL-Cell, Hamburg, Germany) was used for testing all active materials. In the test system, a metal lithium chip as an anode (Reference EQ-Lib-LiC45, MTI Corp, Richmond, CA, USA) and a glass fibre filter paper (Whatman GF/A, Sigma Aldrich, Darmstadt, Germany) as a separator, which was soaked with 1M LiTFSI-TEGDME electrolyte for 5 min, were used. The electrolyte was prepared mixing 1 M bis(trifluoromethane)sulfonimide lithium salt (LiTFSI, Sigma-Aldrich-15224, Darmstadt, Germany) in tetraethylene glycol dimethyl ether (TEGDME, Sigma-Aldrich-172405, Darmstadt, Germany). O_2_-cathodes were prepared by bare coating of a slurry composed of the studied carbons, 5 wt% Polyvinylidene Fluoride (PVDF Kynar ADX 161, Arkema, Colombes, France) as a binder dispersed in 1-methyl-2-pyrrolidinone (NMP, Scharlau-ME05031000, Barcelona, Spain) and carbon black (Timcal Super C65, Timcal Ltd, Bodio, Switzerland) as a conductive additive with a 8:1:1 final dried weight ratio. They were mixed in a glass vial and stirred with an electromagnetic stirring method for at least 4 h and, finally, mixed with a homogenizer (Benchmark Scientific Inc. D1000, Edison, NJ, USA) for at least 10 min. Afterward, the prepared ink was spread onto a gas diffusion layer (GDL, SIGRACET 24 BA, SGL Group, Wiesbaden, Germany). The wet thickness was 100 μm and coating was made with the bar coating method. The coated GDL was dried in an oven at 50 °C overnight under vacuum 0.05 Pa. Subsequently, disk-shaped electrodes were punched with a diameter of 18 mm and an active carbon electrode loading ranged from 3.2 to 4.3 mg/cm^2^. The cell was assembled in an argon-filled glovebox and then connected to oxygen gas circulation (purity grade 4.5–99.995%) through the cathode with a flow rate of 5 mL/min during the experiments.

All electrochemical measurements were performed using a VMP3 and BCS810 multi-channel galvanostat-potentiostat (Bio-Logic SAS, Seyssinet-Pariset, France). A 2.15 and 4.35 V vs. Li/Li^+^ cut-off voltage was used for the galvanostatic cycling tests. The full discharge capacity was measured by discharging the cell at a constant current density of 100 mA/g of active carbon. Then another cell was assembled for the cyclability test and the performance of the material was studied by charging and discharging the battery cell at 50 mA/g with a limited capacity of 300 mAh/g. 

## 3. Results

### 3.1. Determination of Real Amylose Content in Commercially Available Starches

**Figure 1:**
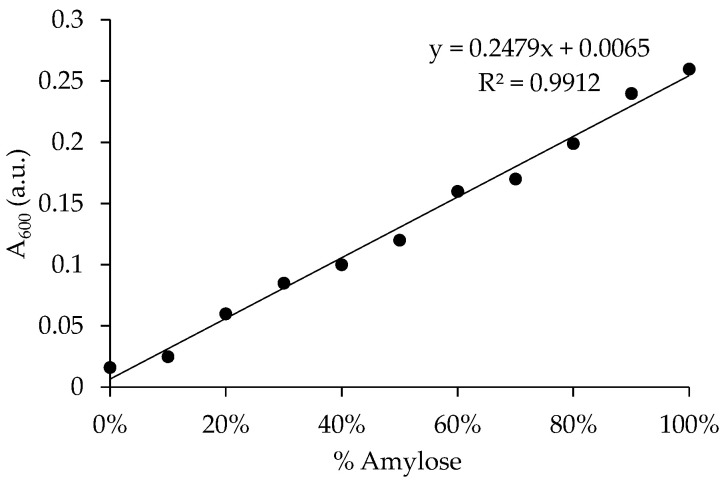
Calibration curve obtained from measurements of absorbance at 600 nm against percentage amylose (w/w) for samples prepared with different contents of pure amylose and amylopectin.

A calibration curve was obtained from the absorbance measurements of the mixtures of pure amylose and amylopectin with iodine, as shown in Figure 1. The real amylose content of commercially available starches was calculated from the equation obtained from this calibration curve (see Table 1).

### 3.2. Preparation of Mesoporous Carbons from a Modified Starbon^®^ Based Process

Mesoporous carbons have been prepared following two different processes based on the Starbon^®^ process with some modifications. The results obtained with each of them are explained in the following sections. 

#### 3.2.1. Modified Starbon^®^ Based Process with a Previous Treatment of the Starches with the IL

In an attempt to control the mesoporosity of the final carbons from the starting starch mixtures and to try to improve it, a pre-treatment of the starch and mixtures with an IL has been introduced before the modified Starbon^®^ based process. It is already known that some IL are good solvents for polysaccharides and can increase its solubilization in comparison with water [24,30]. According to this, the use of an IL can improve the chemical interaction between the starch chain itself and the different starch types.

**Figure 2:**
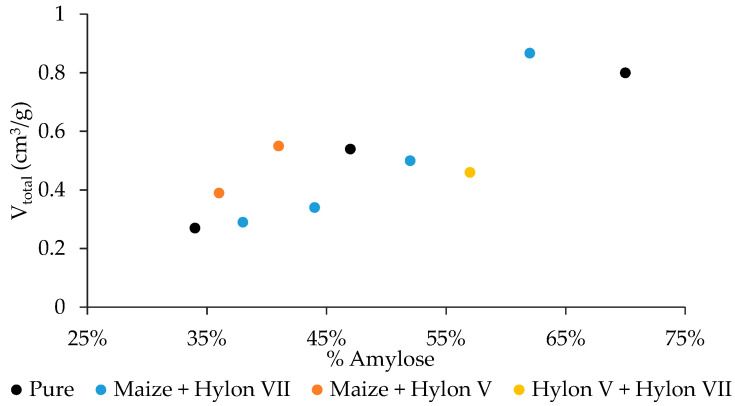
Graphic of the total pore volume against percentage of amylose (*w*/*w*) for different starches and their mixtures, prepared by an IL treatment and a modified Starbon^®^ based process, carbonized at 800 °C.

For this process and as explained in the previous section, the starch mixtures were first blended with an IL and then processed following a modified Starbon^®^ based process. In order to generate carbons with different amylose contents than the commercial ones, various mixtures of Maize, Hylon V. and Hylon VII were prepared and the final nanoporosity was measured (Table 2 and Figures S1–S10 in Supplementary File). An expected tendency of an increasing mesoporosity with greater amylose content was found, but with a higher increase in starch mixtures than in pure starches (Figure 2). The highest total pore volume and mesoporosity was found for the sample prepared from Maize and Hylon VII with a 60 wt% amylose (0.87 cm^3^/g and 80%, respectively), which is higher than pure Hylon VII with a 70 wt% amylose (0.80 cm^3^/g and 77%). However, at lower amylose content (less than 50 wt%), the highest mesoporosity is obtained with the Maize Hylon V mixture, showing that the final mesoporosity not only depends on the amylose content but also on the type of starch. The pore size grows with increasing amylose content and is higher for the mixture of MHV vs. MHVII at the same amylose content.

**Figure 3:**
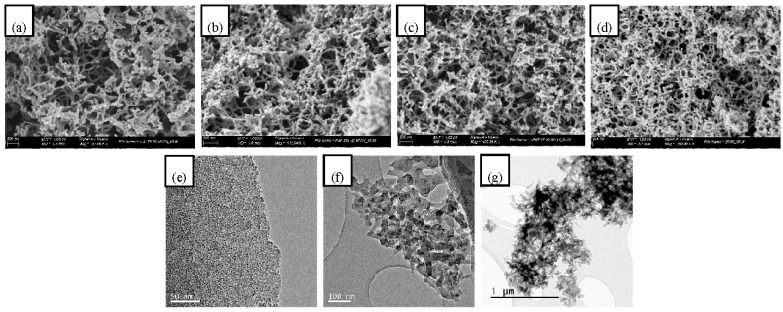
HRSEM images of mesoporous carbons prepared from starch mixtures of Maize and Hylon VII with different contents of amylose: (**a**) 36 wt%, (**b**) 44 wt%, (**c**) 52 wt%, (**d**) 60 wt%, (**e**–**g**) TEM images of mesoporous carbon prepared from MHVII 60 wt% amylose, from higher to lower magnification.

The morphology of the carbons prepared from Maize and HVII with different contents of amylose was studied by HRSEM microscopy. The obtained images in Figure 3a–d) show a nanorod-like carbon structure for all the samples, but with smaller and more homogeneous distributed nanorods in case of the sample with 60 wt% amylose. The morphology of the sample MHVII with a 60 wt% of amylose was also studied by TEM microscopy, showing the same nanorod-like carbon structure (Figure 3e–g).

**Figure 4:**
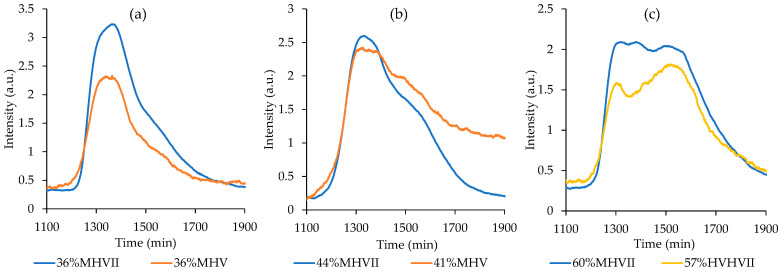
GPC spectra of starch mixtures after IL treatment: (**a**) samples with a 36 wt% of amylose prepared from Maize + Hylon V (orange) and Maize + Hylon VII (blue), (**b**) samples with a 41 and 44 wt% of amylose prepared from Maize + Hylon V (orange) and Maize + Hylon VII (blue), respectively, and (**c**) samples with a 60 and 57 wt% of amylose prepared from Maize + Hylon VII (blue) and Hylon V + Hylon VII (yellow), respectively.

To better understand the higher mesoporosity of some of the mixtures, GPC studies were performed to study the possible influence of molecular weight of the starch mixture on the porosity. For this, GPC analysis was performed on starch mixtures after their treatment with the IL. Figure 4 shows the comparison of GPC spectrum obtained from mixtures of Maize + Hylon V, Maize + Hylon VII, and Hylon V + Hylon VII with similar amylose content but different mesoporosity. The shape of the curve changes when the amylose content varies due to the different content of amylose vs. amylopectin, that have different molecular weight. However, the shape of the curve is similar for different starches with similar amylose content, and the maximum of the curve is the same. This indicates that the mixtures with a similar amylose content have the same molecular weight after treatment with IL, which means that the molecular weight is not responsible for different mesoporosity.

##### Influence of Starch Mixture Concentration in Carbon Mesoporosity

In order to find the optimal concentration of the starch mixture (MHVII 60 wt% amylose) with the IL in the pre-treatment step to obtain the highest mesoporosity, different concentrations were studied. For this, mesoporous carbons were prepared following the previous described procedure with the starch mixture dispersion in IL and after the modified Starbon^®^ based process. According to the results (Table 3 and Figures S11–S14 in Supplementary File), the dispersion formed with a 20 wt% of starch mixture in the IL resulted in the concentration with a higher mesoporosity.

#### 3.2.2. Modified Starbon^®^ Based Process

As a comparison, mesoporous carbons were generated by the modified Starbon^®^ process consisting of a preparation of the mixture of starches in water. Then a gelation with sonication, a solvent elimination by freeze-drying, and finally the carbonization at 800 °C without the IL pre-treatment. The porosity (Table 4 and Figures S15 and S16 in Supplementary File) was measured for a carbon prepared from Maize and Hylon VII (60 wt% amylose) and carbon prepared from Hylon VII (70 wt% amylose). It is clearly seen that the measured mesoporosity is lower than the obtained from the carbons prepared with the IL pre-treatment.

### 3.3. Preparation of Mesoporous Carbons with a “Low-Cost” Process

An alternative route to prepare mesoporous carbons using the IL pre-treatment explained before but without the modified Starbon^®^ based process was studied. For this, the mixed starches were directly carbonized at 800 °C without the gelation and freeze-drying steps. The nanoporosity results obtained using different concentrations are shown in Table 5 and Figures S17–S21 in Supplementary File. The highest mesoporosity is obtained with a 5 and 15 wt% concentration. Although this alternative produces carbons with a lower mesoporosity, it saves time (4 days for the retrogradation and freeze-drying steps) and energy (no need of sonication, refrigeration, and freeze-drying) and, therefore, it is a low-cost alternative to obtain mesoporous carbons.

### 3.4. Recyclability of the IL

With the aim to further diminish the costs of the mesoporous carbon production, the recovery and reuse of the IL was studied. For this, the IL was recovered by evaporation of the ethanol-water used during the washing steps in the previous processes and reused up to three consecutive times. The results of the obtained nanoporous carbons are shown in Table 6 and Figures S22–S25 in Supplementary File. As can be seen, there is a loss of mesoporosity when using the recycled IL. However, after treating the IL with molecular sieves (60% MHVII-IL recycled + MS), the IL was completely dried and re-solidified. This resulted in carbons with a comparable nanoporosity of the carbons produced with non-recycled IL.

To study the IL chemical stability after its use, several analyses were performed (TGA, ^1^HNMR, and FTIR).

**Figure 5:**
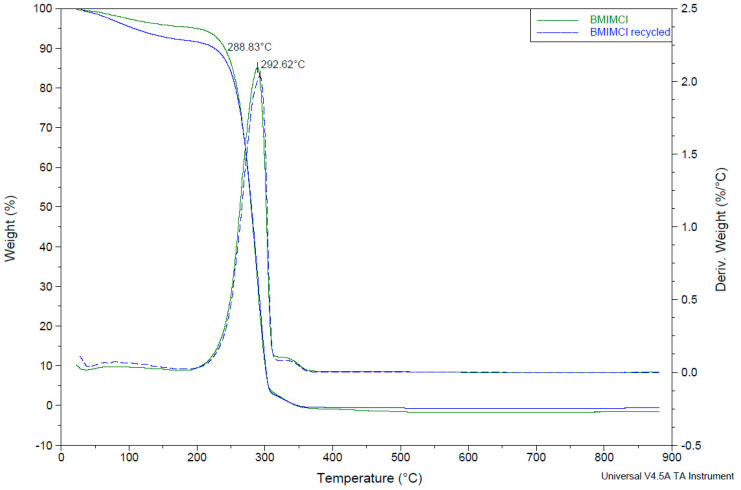
TGA of pure IL (green) and recycled IL (blue) between 0–900 °C represented as continuous lines, and their derivative weight (%/°C) represented as discontinuous lines.

A thermogravimetric analysis was carried out for the pure IL and for the recycled IL obtained after a first recovery. The graphic obtained shows a higher loss of weight between 0–200 °C in the recycled IL due to a higher water content, as can be seen in Figure 5. At 100 °C, a 2.7 wt% weight loss was found for the pure IL, but a 4.8 wt% weight loss was found for the recycled IL. This confirms that, despite the evaporation, there is still some water in the IL structure.

**Figure 6:**
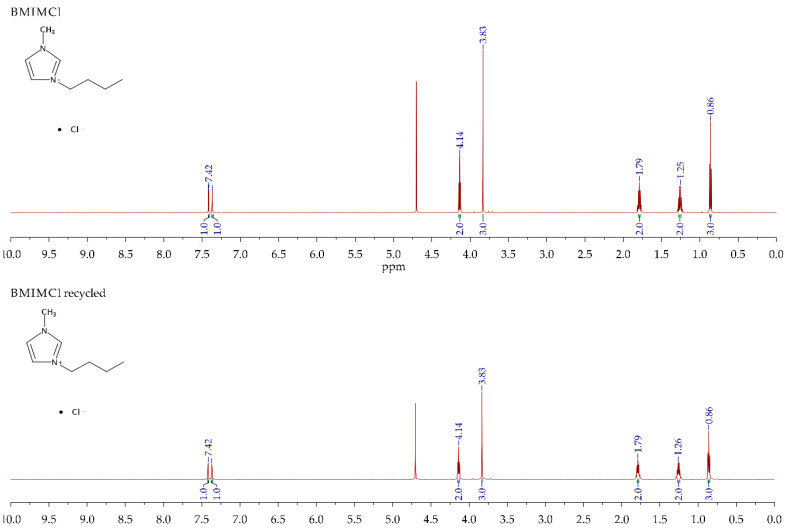
^1^HNMR spectra of pure IL (**top**) and recycled IL (**bottom**).

The structure of pure IL and recycled IL was also studied by ^1^H NMR (Figure 6). All the expected peaks of the molecule can be observed before and after its use, confirming that there are no changes in the molecular structure of the IL after its use. 

**Figure 7:**
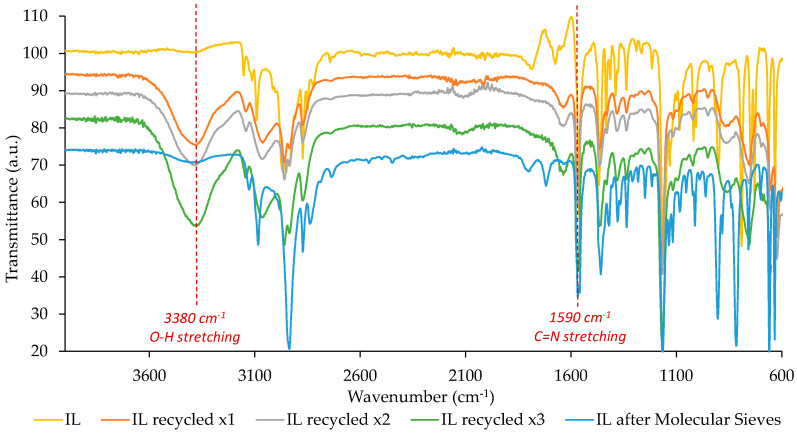
FTIR-ATR spectra of pure IL (yellow line), recycled IL (x1 orange line, x2 grey line, x3 green line), and recycled and treated with molecular sieves IL (blue line).

These results were also confirmed by FTIR. The structure of the recycled IL obtained after every recovery was analysed by FTIR-ATR spectroscopy and the obtained spectra are shown in Figure 7. There are no important changes in the molecular structure, and, therefore, it is confirmed that the IL chemical structure was not modified after its use for the starch mixture dispersion.

### 3.5. Preparation of Mesoporous Carbon to be Used in Li-O_2_ Battery Cells Studies

For the Li-O_2_ battery cells studies, different mesoporous carbons were prepared from starch and chitosan. In case of starch, the prepared samples followed the previous process described in Section 2.3.2 with Maize + Hylon VII at different amylose content with the IL pre-treatment and the modified Starbon^®^ based process. For the chitosan precursors, a modified Starbon^®^ based process was followed, as described in Section 2.6. All samples were carbonized at 1000 °C instead of 800 °C to attempt to increase the electrical conductivity of the carbons, and then to improve the kinetics of the battery reactions. 

The nanoporosity data can be seen in Table 7 and Figures S26–S30 in Supplementary File in comparison with the reference material used in the Li-O_2_ studies (Timcal). The mesoporosity increases for the MHVII samples as the amylose content increases, as expected. A high content of nitrogen (5.5 wt%) was measured for the chitosan-derived carbon. The reference sample (Timcal) has a low mesoporosity.

**Figure 8:**
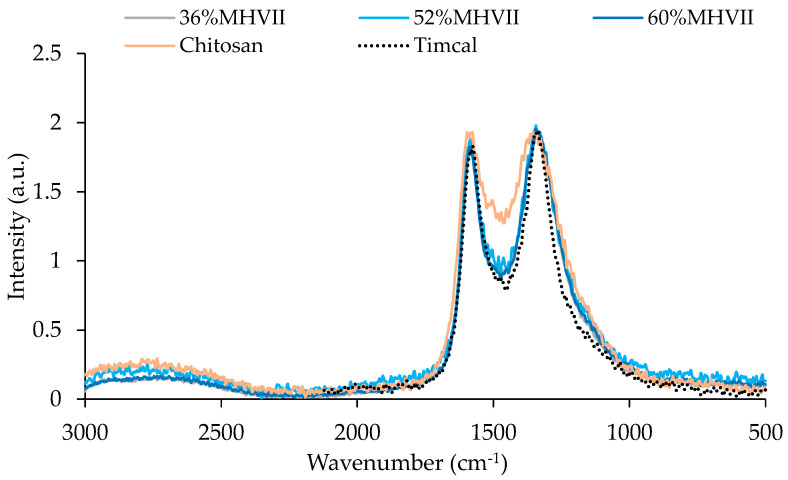
Raman spectra of all carbons prepared for the Li-O_2_ battery cell studies.

Raman spectra of the carbons were investigated to estimate the degree of graphitization of the carbons (Figure 8). The disorder of the graphitized carbon was estimated by the intensity ratio between the D band (disordered graphite) and G band (ordered graphite), I_D_/I_G_ (Table 7), showing no relevant differences.

### 3.6. Application of Mesoporous Carbon as Cathodic Material in Li-O_2_ Battery Cells

**Figure 9:**
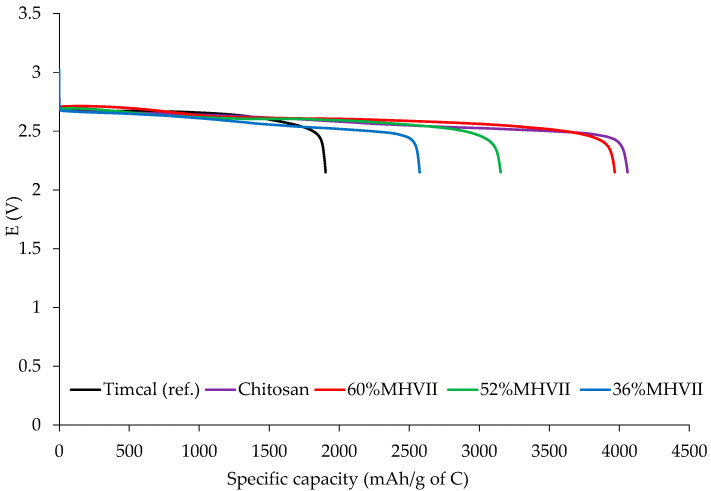
Discharge capacities for the different carbons.

**Figure 10:**
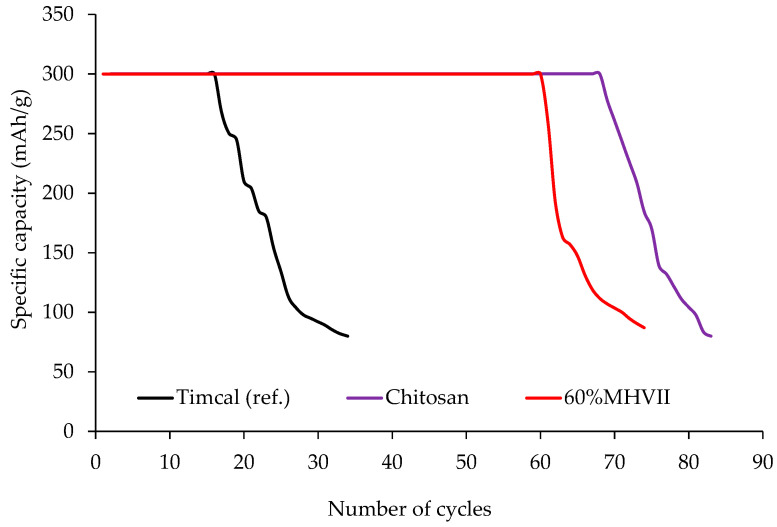
Cycling results for the Li-O_2_ batteries with the prepared carbons.

The mesoporous carbons produced in the previous section were tested in the Li-O_2_ cell and compared to the material reference (Timcal). The full discharge capacity was measured by discharging the cell at 100 mA/g. Then the cyclability of the cell is studied by charging and discharging the battery cells at 50 mA/g with a limited capacity of 300 mAh/g. The results are shown in Table 8, Figure 9 and Figure 10. It is demonstrated that the carbons with a higher mesoporosity show a higher specific discharge capacity except for the case of chitosan-derived carbons, in which the N-doping improves the performance. The cycling studies also demonstrate the same tendency and the carbons with higher mesoporosity or N-doped (60%MHVII and Chitosan, respectively) present a higher cycling than the reference material (Timcal). 

## 4. Discussion

Previous studies demonstrated the influence of amylose content on the final carbon mesoporosity after a Starbon^®^ based process. In this sense, the starch with a higher amylose content, for instance Hylon VII with 70 wt% amylose, produced carbons with a higher mesoporosity. In this study, we demonstrated that, apart from amylose content, the type of starch mixture influences the final meso-porosity. For that, we applied a pre-treatment of the starch mixtures with an IL followed by a modified Starbon^®^ process with sonication for the gelation step instead of a microwave treatment. We showed that, at high amylose content (higher than 50 wt%), the highest mesoporosity was obtained at 60 wt% amylose using a mixture of Maize and Hylon VII instead of the pure Hylon VII with a higher amylose content (70 wt% amylose). At lower amylose content (less than 50 wt%), the best mixture to increase the mesoporosity of the carbon was the mixture of Maize and Hylon V. This result was also confirmed by the HRSEM images of the mixtures Maize and Hylon VII, where the nanorod-like carbon structures forming the mesopores became smaller and more homogeneously distributed through the material when the amylose content was increased (see Figure 3). These results indicate that the treatment of the starch mixture with the IL influences the starch structure that becomes more organized, creating more pores in the mesopore range. Since the GPC studies did not show any relevant difference between the different starch mixtures with similar amylose content, future studies based on the characterization of the three-dimensional structure and non-covalent interactions would better explain these results.

In this work, we also demonstrate the effect of the process used on the final carbon mesoporosity. For this, we compared the modified Starbon^®^ based process (with sonication) with and without the IL pre-treatment (IL + Starbon^®^ and Starbon^®^, respectively, in Table 9 and Figure S31 in Supplementary File) and the process with the direct carbonization of the IL pre-treated starches (IL in Table 9). It is clearly seen that the IL pre-treatment increases the mesoporosity of the final carbons. The higher mesoporosity was found by an IL pre-treatment followed by the modified Starbon^®^ based process (0.87 cm^3^/g for 60% MHVII and 0.80 cm^3^/g for 70% HVII), but a more cost effective process was achieved by direct carbonization of the IL-treated starches (0.33 cm^3^/g for 60% MHVII and 0.44 cm^3^/g for 70% HVII) with a higher mesoporosity than the carbons obtained directly by a modified Starbon^®^ based process (0.25 cm^3^/g for 60% MHVII and 0.23 cm^3^/g for 70% HVII).

Best performing materials in Li-O_2_ battery cells were Chitosan and 60% MHVII with 4057 mAh/g and 3978 mAh/g specific capacity and 69 and 60 cycles, respectively, without any capacity lost. When compared to the reference material (Timcal), we see that the capacity was doubled and the cyclability of the cell increased more than four times. From these results, it is demonstrated that, for Li-O_2_ technology, the total carbon mesoporosity contributed highly on the performance of the materials for both specific capacity and number of cycles. For the carbons obtained from MHVII, the one with the highest mesoporosity (60%MHVII) has the highest capacity and the one with the lowest mesoporosity (36%MHVII) has the lowest capacity. This shows the importance of the mesoporosity in Li-O_2_ technology, as it enables a better oxygen diffusion and lithium transfer [26] and, therefore, an enhanced Li_2_O_2_ formation, which is directly related to the capacity. However, in the case of the carbon obtained from chitosan, although the mesoporosity is lower, the capacity was the highest achieved. In this case, the effect of the presence of nitrogen clearly improves the performance of the material, as already described in the literature [31,32,33,34].

## 5. Conclusions

This work describes new processes to produce carbon materials from polysaccharides with controlled mesoporosity consisting in the IL pre-treatment of starch or starch mixtures. For the fabrication of high mesoporous carbons, this IL pre-treatment is followed by a modified Starbon^®^ based process, using sonication for the gelation and followed by retrogradation, freeze-drying, and carbonization under inert conditions. A low-cost process to produce mesoporous carbons directly from the carbonization of the IL-treated starches is also shown. Different starches, starch mixtures, and different concentrations were tested to find the optimal conditions that resulted in a carbon material with an increased mesoporosity. The highest mesoporosity was found for the mixture of starch from maize (Maize) and corn (Hylon VII) containing a 60 wt% amylose and prepared with a concentration of 20 wt% in the IL (V_pore total_ = 0.87 cm^3^/g). For all these methods, it was demonstrated that the used IL can be recovered and reused without any effect on the final mesoporosity. Moreover, an N-doped mesoporous carbon was obtained from chitosan following a similar Starbon^®^ based process.

These carbons were tested as cathode components in Li-O_2_ batteries showing an increased capacity and cycling stability with the carbons with the higher mesoporosity. The doping with nitrogen, in the case of the chitosan-derived carbon, showed a further increased performance. 

## 6. Patents

Process for preparing mesoporous carbon material. LEITAT Submitted 28/02/2019 Patent nr. P20382135.0.

## Figures and Tables

**Table 1:** Real amylose content of commercially available starches from Ingredion.

Starch	wt % Real Amylose
Maize	34%
Hylon V	47%
Hylon VII	70%

**Table 2:** Nanoporosity data of the prepared mesoporous carbons at 800 °C.

Precursor	Weight Ratio	wt% Amylose	S.A._BET_ (m^2^/g)	V_total_ (cm^3^/g)	% Mesoporosity	BJH Pore Diameter (nm)
Maize	1	34%	415	0.27	52%	11
HV	1	47%	458	0.54	70%	17
HVII	1	70%	547	0.80	77%	20
36% MHVII	1:0.07	36%	424	0.29	53%	11
44% MHVII	1:0.39	44%	370	0.34	98%	16
52% MHVII	1:1	52%	484	0.50	74%	25
60% MHVII	1:2.55	60%	536	0.87	80%	22
36% MHV	1:0.15	36%	470	0.39	61%	15
41% MHV	1:1.30	41%	475	0.55	75%	23
57% HVHVII	1:0.77	57%	458	0.46	72%	15

**Table 3:** Nano-porosity data of carbons obtained from Maize + Hylon VII (60 wt% amylose) at different concentrations.

Precursor	wt% Starch Mixture in IL	S.A._BET_ (m^2^/g)	V_total_ (cm^3^/g)	% Mesoporosity	BJH Pore Diameter (nm)
60% MHVII 5	5	449	0.52	69	17
60% MHVII 10	10	448	0.42	60	16
60% MHVII 15	15	459	0.57	70	21
60% MHVII 20	20	536	0.87	80	22
60% MHVII 25	25	475	0.78	79	24

**Table 4:** Nanoporosity data of mesoporous carbons obtained from the modified Starbon^®^ process.

Precursor	Weight Ratio	wt% Amylose	S.A._BET_ (m^2^/g)	V_total_ (cm^3^/g)	% Mesoporosity	BJH Pore Diameter (nm)
60%MHVII no IL	1:2.55	60%	398	0.25	31	10
70%HVII no IL	1	70%	395	0.21	13	6

**Table 5:** Nanoporosity data of carbons obtained from Maize + Hylon VII (60 wt% amylose) prepared with the “low-cost” alternative route at 800 °C.

Precursor	wt% Starch Mixture in IL	S.A._BET_ (m^2^/g)	V_total_ (cm^3^/g)	% Mesoporosity	BJH Pore Diameter (nm)
60% MHVII-LC 5	5	359	0.51	77	14
60% MHVII-LC 10	10	410	0.37	51	17
60% MHVII-LC 15	15	441	0.56	70	19
60% MHVII-LC 20	20	335	0.33	59	14
60% MHVII-LC 25	25	412	0.38	55	13

**Table 6:** Nanoporosity data of carbons obtained from Maize + Hylon VII (60 wt% amylose) with pure and recycled ionic liquid (IL).

Precursor	S.A._BET_ (m^2^/g)	V_total_ (cm^3^/g)	% Mesoporosity	BJH Pore Diameter (nm)
60% MHVII	536	0.87	80	22
60% MHVII-IL recycled x1	490	0.59	67	23
60% MHVII-IL recycled x2	486	0.49	63	14
60% MHVII-IL recycled x3	417	0.52	70	23
60% MHVII-IL recycled + MS	469	0.82	81	23

**Table 7:** Nanoporosity data, nitrogen content, and I_D_/I_G_ of carbons obtained from starch and chitosan, carbonized at 1000 °C.

Precursor	S.A._BET_ (m^2^/g)	V_total_ (cm^3^/g)	% Mesoporosity	BJH Pore Diameter (nm)	wt% N	I_D_/I_G_
Chitosan 1000	217	0.45	96	14	5.50	1.00
60% MHVII 1000	554	0.71	71	21	-	1.02
52% MHVII 1000	541	0.64	69	17	-	1.00
36% MHVII 1000	529	0.54	61	17	-	1.03
Timcal (ref.)	67	0.19	100	14	-	1.01

**Table 8:** Electrochemical characterization of the mesoporous carbons produced from the different polysaccharides, carbonized at 1000 °C.

Precursor	Specific Discharge Capacity (mAh/g of C)	Total Cycles	Active Material (mg)
Timcal (ref.)	1997	14	3.14
Chitosan 1000	4057	69	3.36
60% MHVII 1000	3978	60	3.80
52% MHVII 1000	3157	-	3.82
36% MHVII 1000	2570	-	4.12

**Table 9:** Nanoporosity data of carbons obtained from Maize + Hylon VII (60 wt% amylose) and from Hylon VII (70 wt% amylose) prepared by three different processes, calcinated at 800 °C.

Precursor	Method	S.A._BET_ (m^2^/g)	V_total_ (cm^3^/g)	% Mesoporosity	BJH Pore Diameter (nm)
60% MHVII	IL + Starbon^®^	536	0.87	80	22
	IL	335	0.33	59	14
	Starbon^®^	398	0.25	31	10
70% HVII	IL + Starbon^®^	547	0.80	77	20
	IL	511	0.44	59	16
	Starbon^®^	395	0.21	13	6

## Supplementary Materials

The following are available online at https://www.mdpi.com/2079-4991/10/10/2036/s1. Figure S1–S31: Nitrogen sorption isotherms (a) and BJH pore size distributions (b) of all produced carbons.
