# Supplementary material for: Mesoporous Carbons from Polysaccharides and Their Use in Li-O2 Batteries
Source: Nanomaterials (Basel). 2020 Oct 15;10(10):2036. doi: 10.3390/nano10102036 (PMC7602621; doi:10.3390/nano10102036)
Supplement: Supplementary file 1 [file nanomaterials-10-02036-s001.pdf]

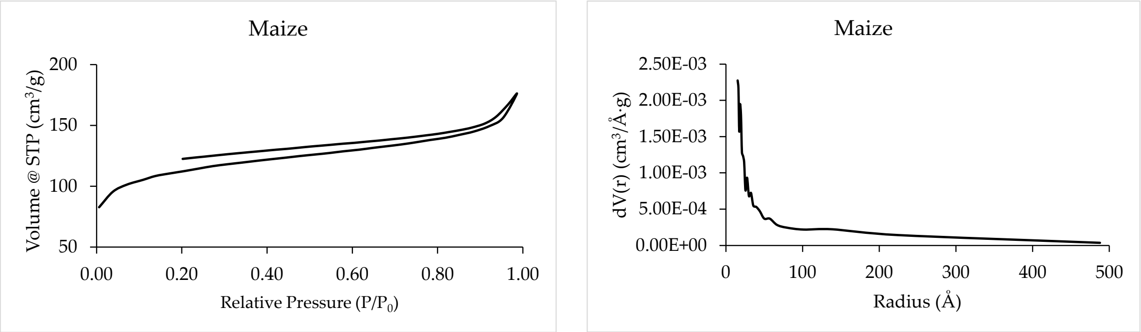

(a) (b)

Figure S1. Nitrogen sorption isotherm (**a**) and BJH pore size distribution (**b**) of Maize.

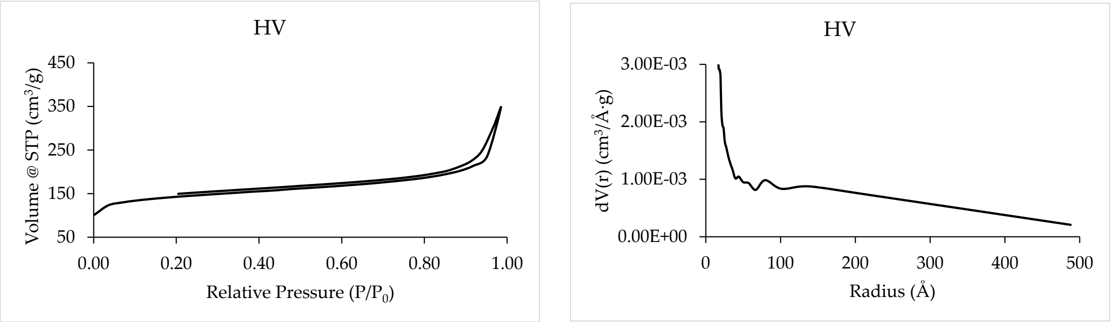

(a) (b)

Figure S2. Nitrogen sorption isotherm (**a**) and BJH pore size distribution (**b**) of HV.

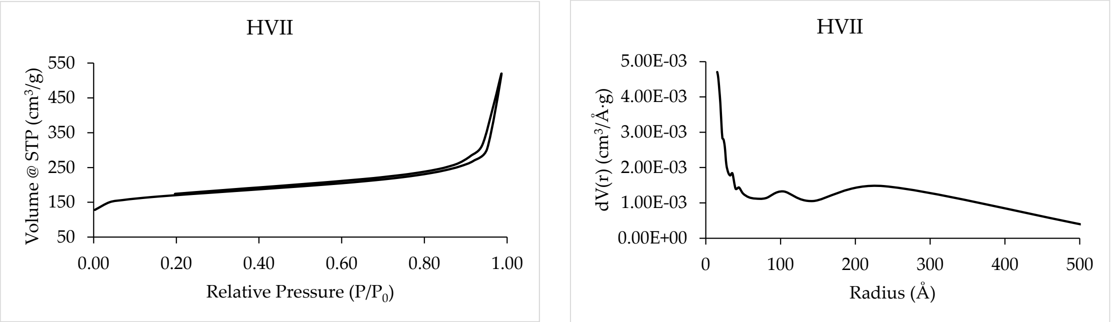

(a) (b)

Figure S3. Nitrogen sorption isotherm (**a**) and BJH pore size distribution (**b**) of HVII.

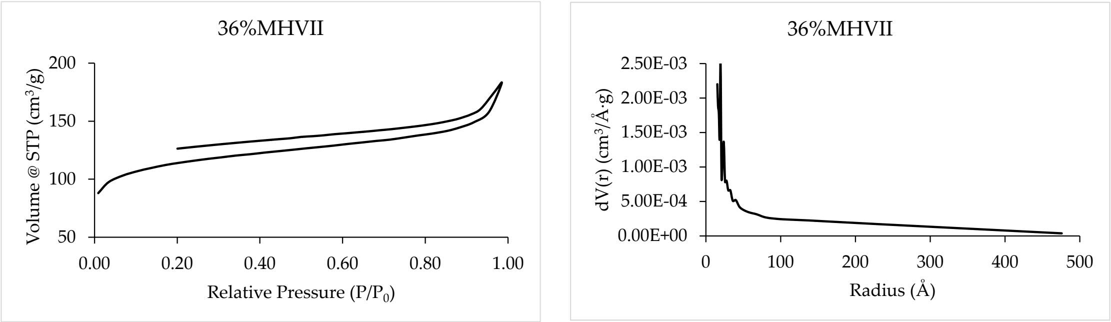

(a) (b)

Figure S4. Nitrogen sorption isotherm (**a**) and BJH pore size distribution (**b**) of 36%MHVII.

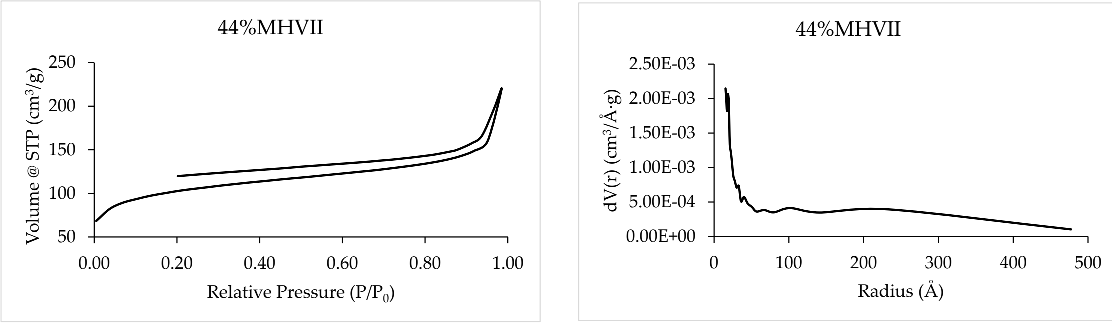

(a) (b)

Figure S5. Nitrogen sorption isotherm (**a**) and BJH pore size distribution (**b**) of 44%MHVII.

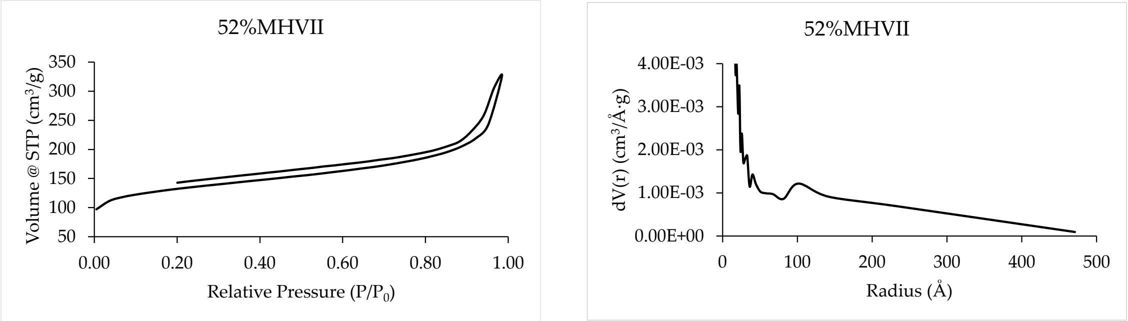

(a) (b)

Figure S6. Nitrogen sorption isotherm (**a**) and BJH pore size distribution (**b**) of 52%MHVII.

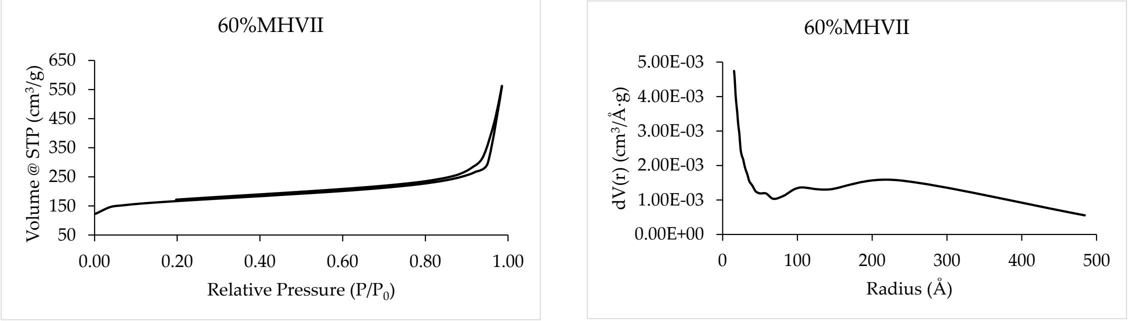

(a) (b)

Figure S7. Nitrogen sorption isotherm (**a**) and BJH pore size distribution (**b**) of 60%MHVII.

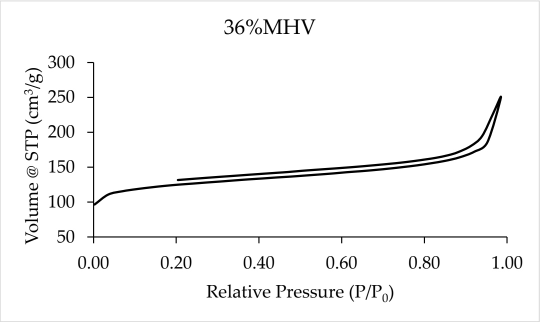

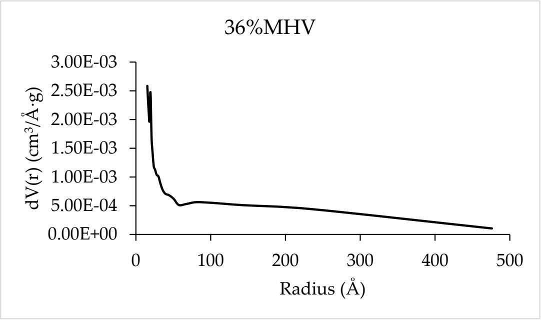

(a) (b)

Figure S8. Nitrogen sorption isotherm (**a**) and BJH pore size distribution (**b**) of 36%MHV.

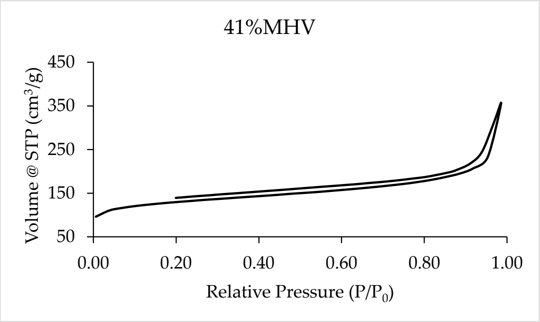

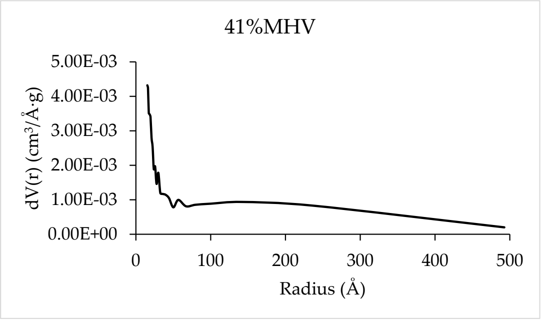

(a) (b)

Figure S9. Nitrogen sorption isotherm (**a**) and BJH pore size distribution (**b**) of 41%MHV.

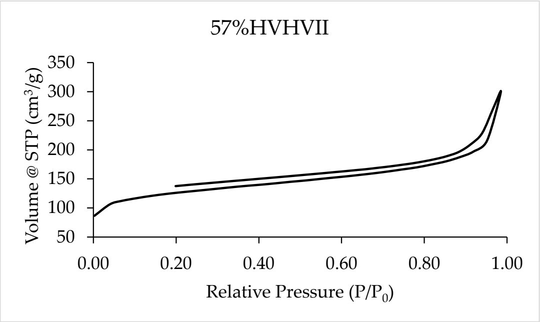

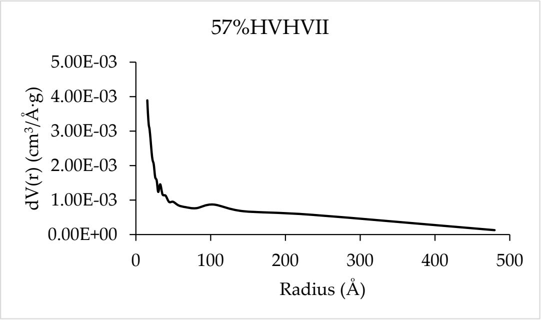

(a) (b)

Figure S10. Nitrogen sorption isotherm (**a**) and BJH pore size distribution (**b**) of 57%HVHVII.

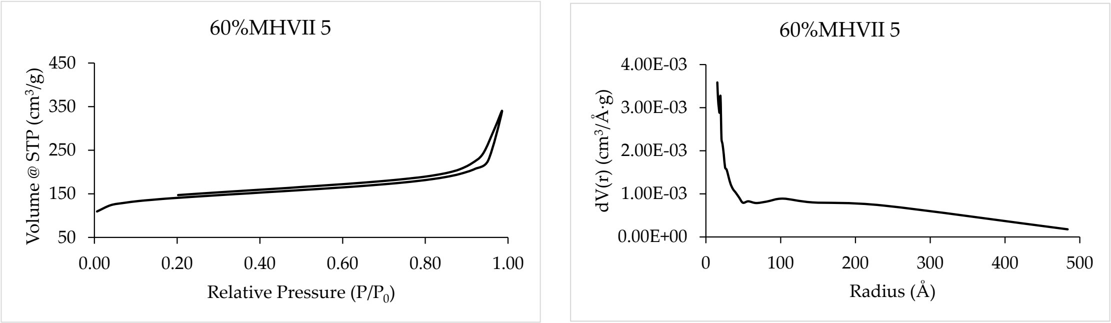

(a) (b)

Figure S11. Nitrogen sorption isotherm (**a**) and BJH pore size distribution (**b**) of 60%MHVII 5.

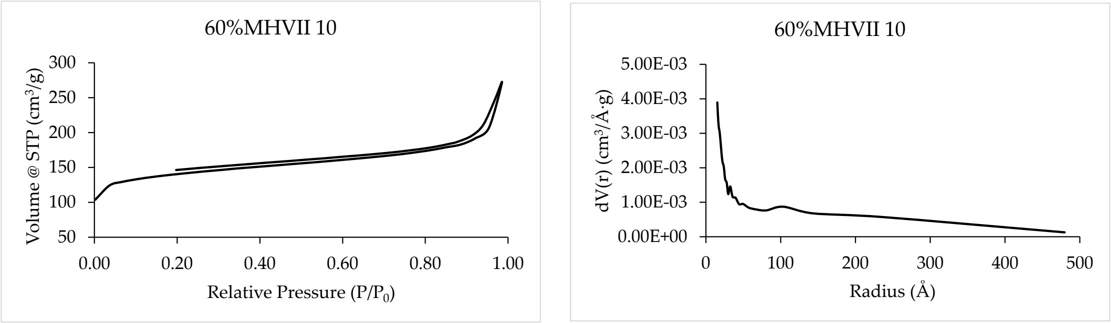

(a) (b)

Figure S12. Nitrogen sorption isotherm (**a**) and BJH pore size distribution (**b**) of 60%MHVII 10.

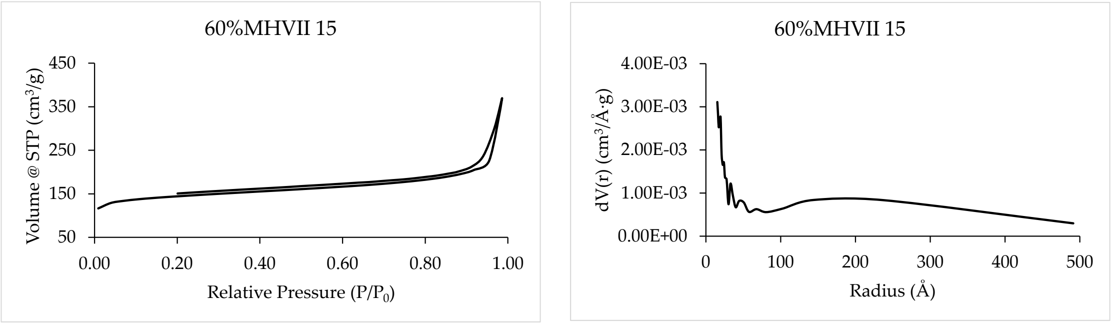

(a) (b)

Figure S13. Nitrogen sorption isotherm (**a**) and BJH pore size distribution (**b**) of 60%MHVII 15.

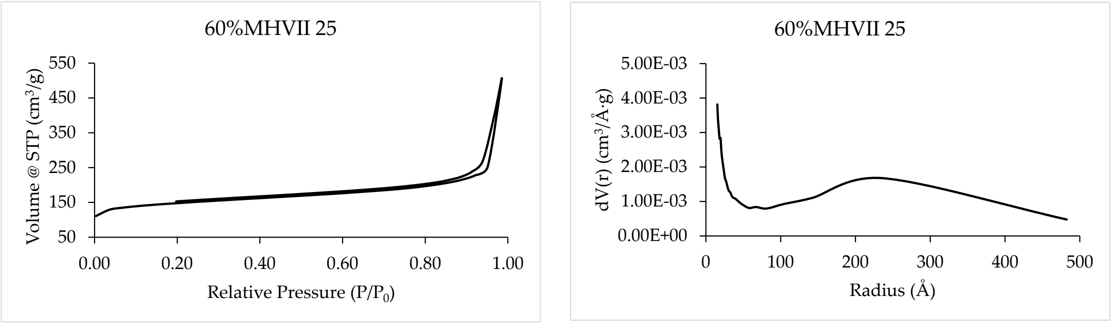

(a) (b)

Figure S14. Nitrogen sorption isotherm (**a**) and BJH pore size distribution (**b**) of 60%MHVII 25.

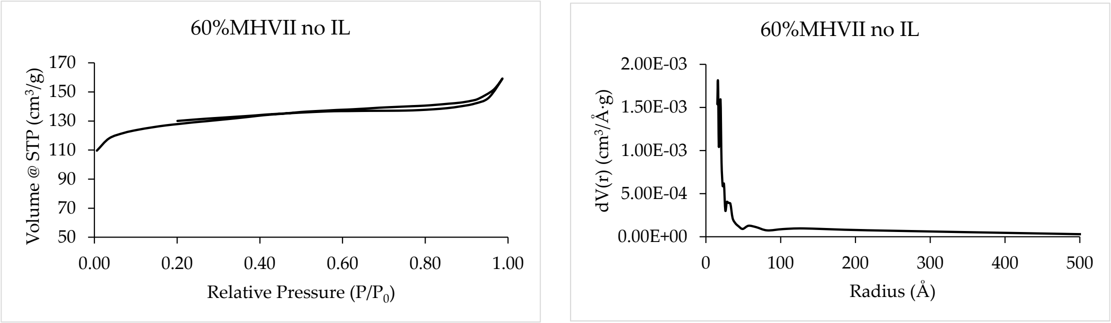

(a) (b)

Figure S15. Nitrogen sorption isotherm (**a**) and BJH pore size distribution (**b**) of 60%MHVII no IL.

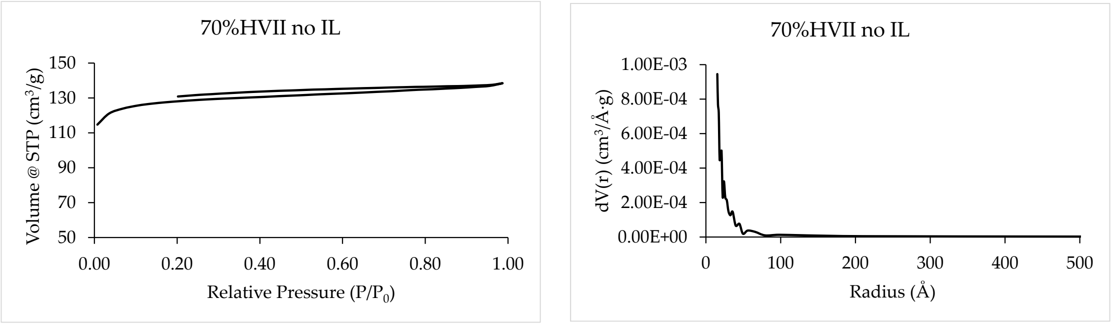

(a) (b)

Figure S16. Nitrogen sorption isotherm (**a**) and BJH pore size distribution (**b**) of 70%HVII no IL.

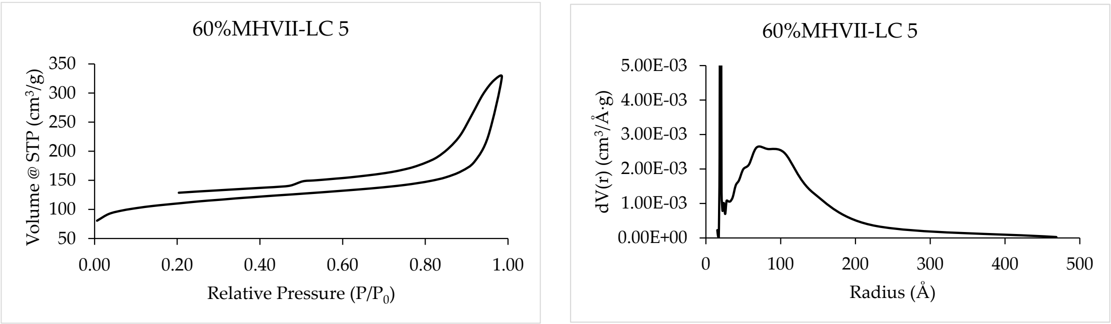

(a) (b)

Figure S17. Nitrogen sorption isotherm (**a**) and BJH pore size distribution (**b**) of 60%MHVII-LC 5.

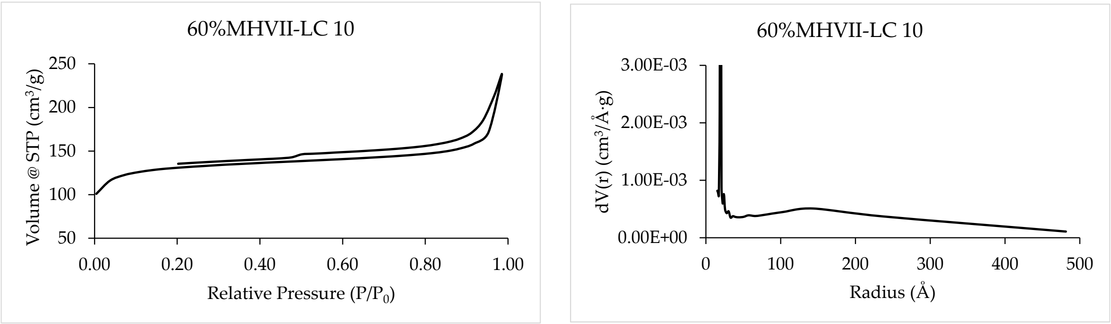

(a) (b)

Figure S18. Nitrogen sorption isotherm (**a**) and BJH pore size distribution (**b**) of 60%MHVII-LC 10.

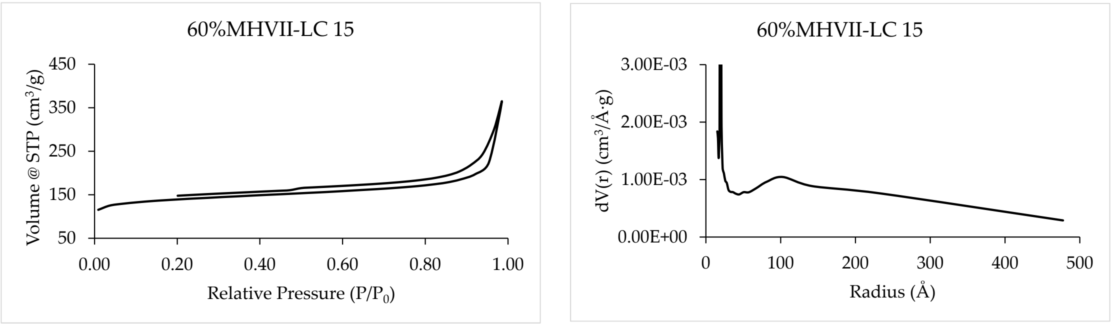

(a) (b)

Figure S19. Nitrogen sorption isotherm (**a**) and BJH pore size distribution (**b**) of 60%MHVII-LC 15.

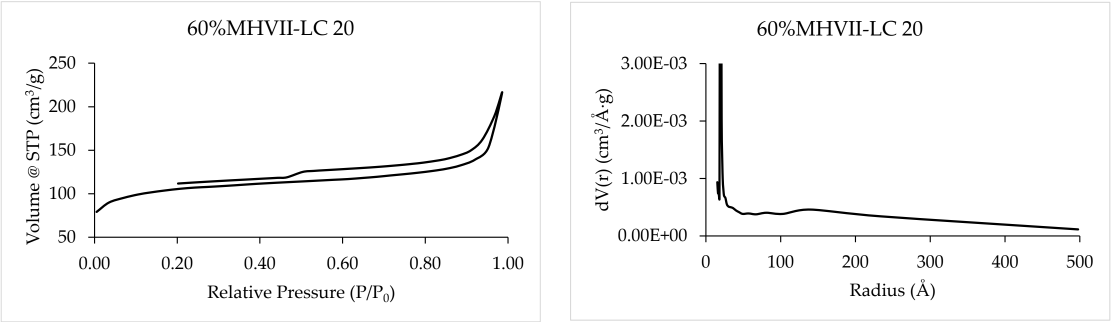

(a) (b)

Figure S20. Nitrogen sorption isotherm (**a**) and BJH pore size distribution (**b**) of 60%MHVII-LC 20.

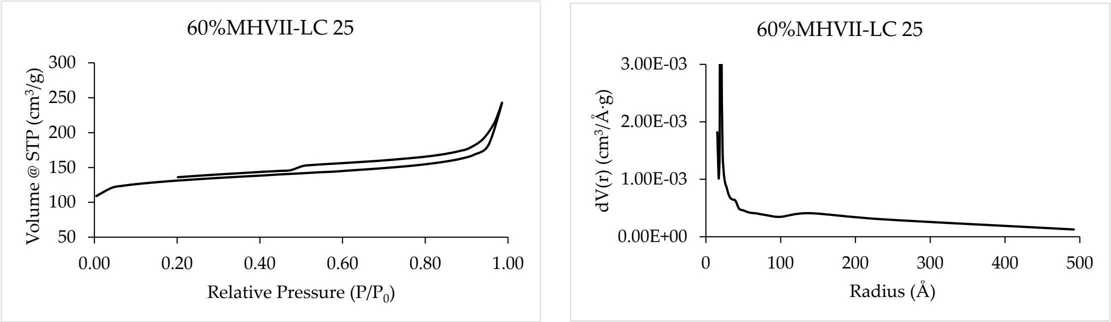

(a) (b)

Figure S21. Nitrogen sorption isotherm (**a**) and BJH pore size distribution (**b**) of 60%MHVII-LC 25.

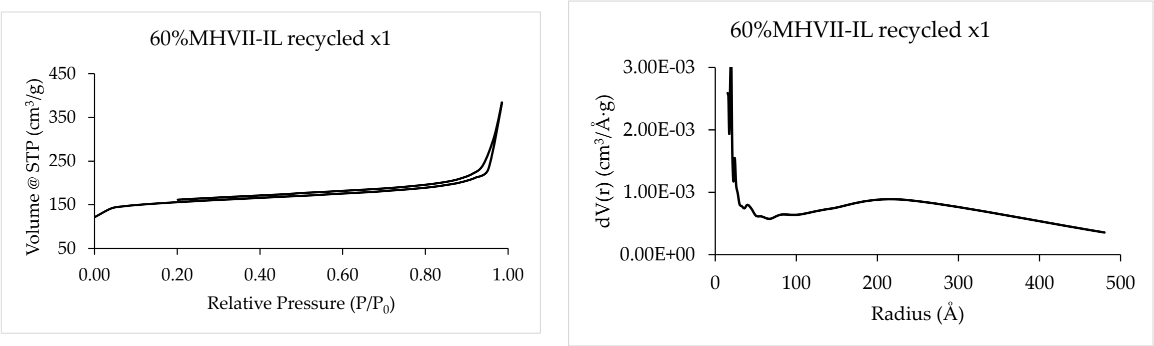

(a) (b)

Figure S22. Nitrogen sorption isotherm (**a**) and BJH pore size distribution (**b**) of 60%MHVII-IL recycled x1.

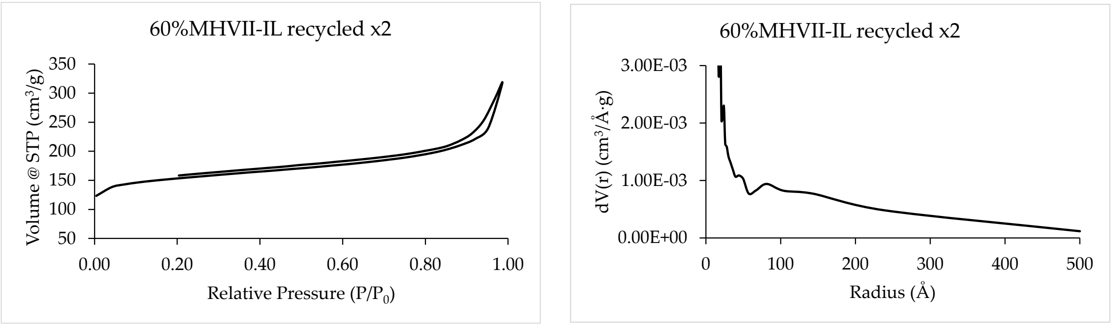

(a) (b)

Figure S23. Nitrogen sorption isotherm (**a**) and BJH pore size distribution (**b**) of 60%MHVII-IL recycled x2.

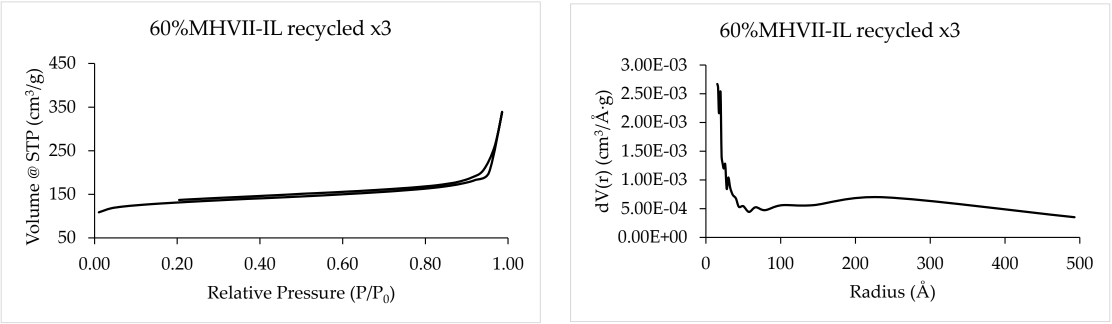

(a) (b)

Figure S24. Nitrogen sorption isotherm (**a**) and BJH pore size distribution (**b**) of 60%MHVII-IL recycled x3.

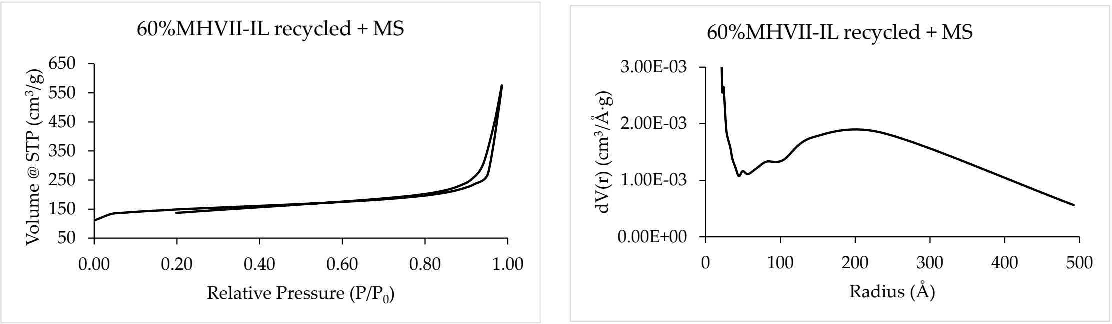

(a) (b)

Figure S25. Nitrogen sorption isotherm (**a**) and BJH pore size distribution (**b**) of 60%MHVII-IL recycled + MS.

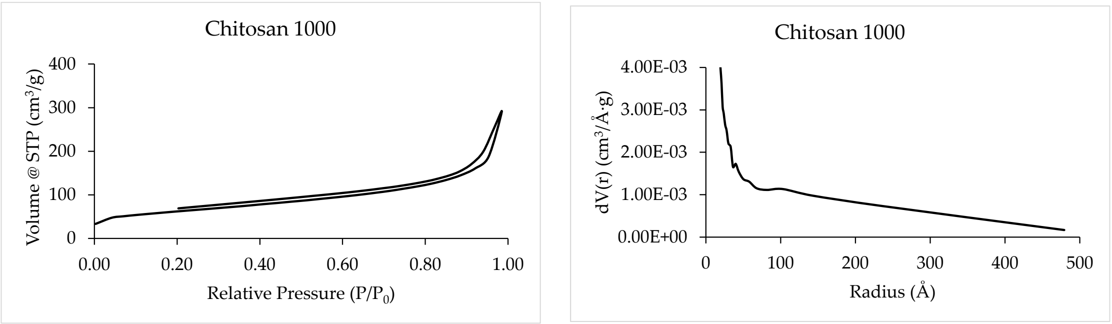

(a) (b)

Figure S26. Nitrogen sorption isotherm (**a**) and BJH pore size distribution (**b**) of Chitosan 1000.

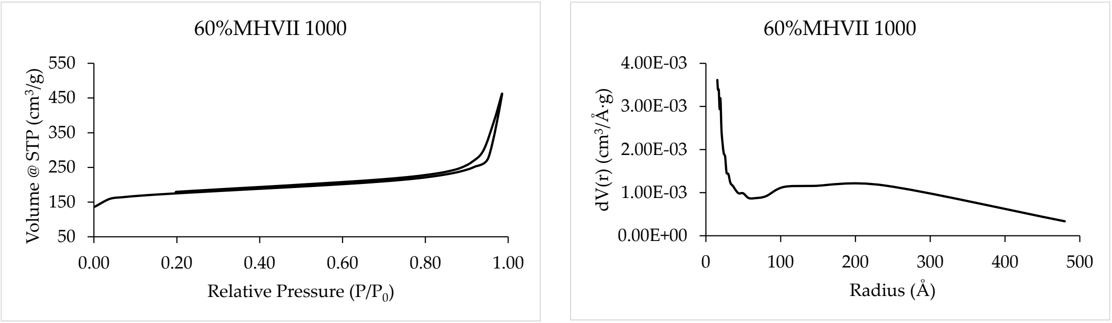

(a) (b)

Figure S27. Nitrogen sorption isotherm (**a**) and BJH pore size distribution (**b**) of 60%MHVII 1000.

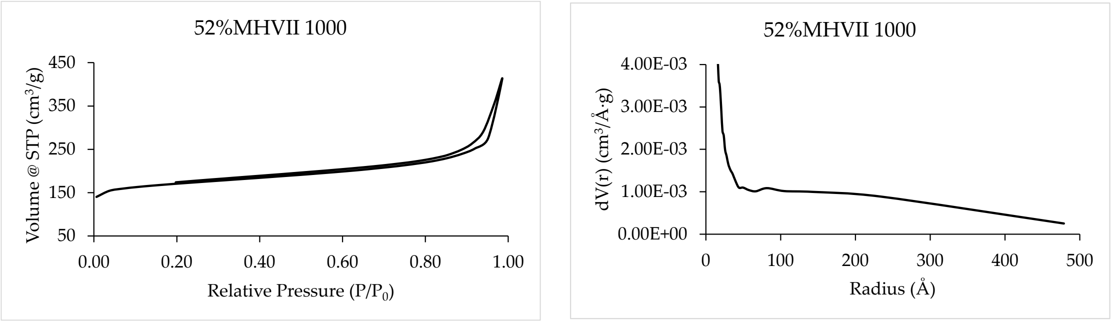

(a) (b)

Figure S28. Nitrogen sorption isotherm (**a**) and BJH pore size distribution (**b**) of 52%MHVII 1000.

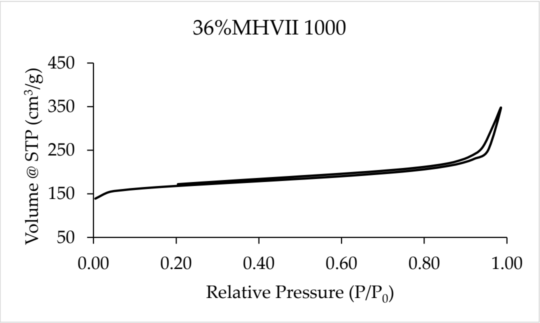

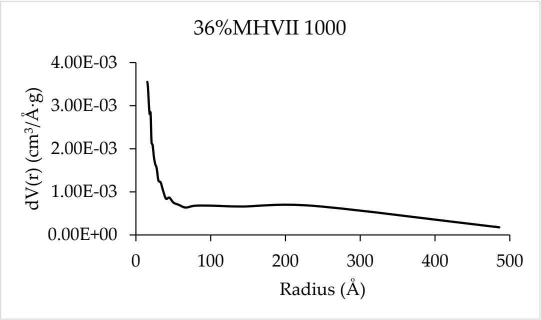

(a) (b)

Figure S29. Nitrogen sorption isotherm (**a**) and BJH pore size distribution (b) of 36%MHVII 1000.

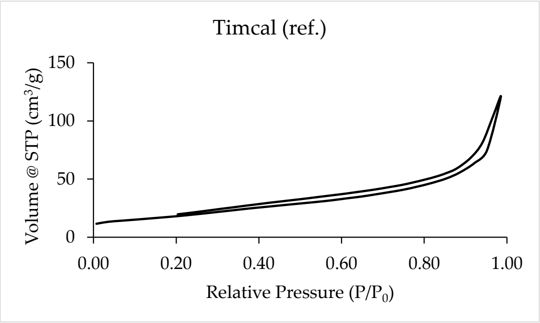

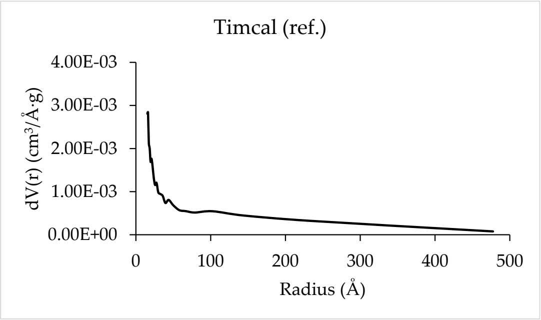

(a) (b)

Figure S30. Nitrogen sorption isotherm (**a**) and BJH pore size distribution (**b**) of Timcal (ref.).

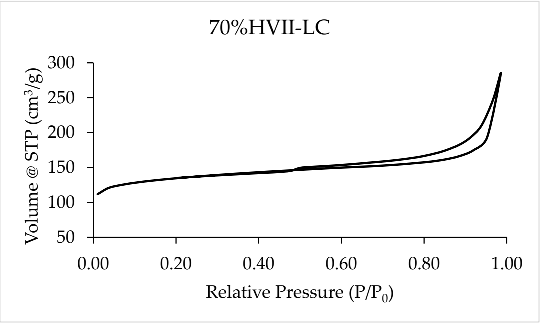

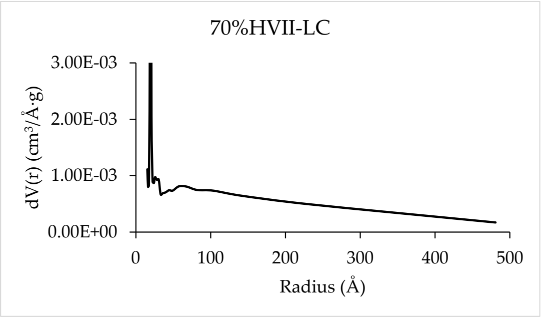

(a) (b)

Figure S31. Nitrogen sorption isotherm (**a**) **a**nd BJH pore size distribution (**b**) of 70%HVII-LC.
